# Vascular Oxidative Stress: Impact and Therapeutic Approaches

**DOI:** 10.3389/fphys.2018.01668

**Published:** 2018-12-04

**Authors:** Cristina M. Sena, Adriana Leandro, Lara Azul, Raquel Seiça, George Perry

**Affiliations:** ^1^Institute of Physiology, Institute for Clinical and Biomedical Research, Faculty of Medicine, University of Coimbra, Coimbra, Portugal; ^2^College of Sciences, One UTSA Circle, University of Texas at San Antonio, San Antonio, TX, United States

**Keywords:** oxidative stress, vasculature, reactive oxygen species, therapies, antioxidants

## Abstract

Oxidative stress has been defined as an imbalance between oxidants and antioxidants and more recently as a disruption of redox signaling and control. It is generally accepted that oxidative stress can lead to cell and tissue injury having a fundamental role in vascular dysfunction. Physiologically, reactive oxygen species (ROS) control vascular function by modulating various redox-sensitive signaling pathways. In vascular disorders, oxidative stress instigates endothelial dysfunction and inflammation, affecting several cells in the vascular wall. Vascular ROS are derived from multiple sources herein discussed, which are prime targets for therapeutic development. This review focuses on oxidative stress in vascular physiopathology and highlights different strategies to inhibit ROS production.

## Introduction

Oxidative stress is an important underlying factor in health and disease. It is originated by an alteration in the balance of reactive oxygen species (ROS) production and antioxidant defense mechanisms (Halliwell, 1994; Betteridge, 2000). Homeostatic ROS concentrations play a crucial role as secondary messengers in many intracellular signaling pathways in both innate and adaptive immune responses (Schieber and Chandel, 2014; Sies, 2017). The problem occurs when ROS bioavailability overtakes the antioxidant defenses. In these conditions, ROS act as destructive agents affecting proteins, lipids and DNA, leading to cellular damage, tissue injury, and inflammation (Halliwell and Gutteridge, 1999). On the other hand, from a mechanistic point of view, oxidative stress may be better defined as a disruption of redox signaling and control. This concept could redirect research to identify crucial perturbations of redox signaling and control and lead to new treatments for oxidative stress-related disease processes (Jones, 2006).

Oxidative stress has been associated with the pathogenesis of several chronic disorders such as neurodegenerative diseases, diabetes, hypercholesterolemia, and atherosclerosis and is a contributory pathogenic factor in obesity-related disorders (Sayre et al., 2001; Schleicher and Friess, 2007; DeMarco et al., 2010; Giacco and Brownlee, 2010; Sena et al., 2013; Bhatti et al., 2017). In addition, vascular oxidative stress is a leading cause in cardiovascular diseases (Li et al., 2014; Förstermann et al., 2017). Elevated oxidative stress leads to vasoconstriction, vascular remodeling, inflammation, and fibrosis (Rodriguez-Porcel et al., 2017). Insufficient cellular protection against oxidative stress has been described as a major factor for the development of vascular diseases (Li et al., 2014). Oxidative stress is also the principal cause of epigenetic changes that occur during aging (Guillaumet-Adkins et al., 2017).

The involvement of reactive oxygen and nitrogen species has been extensively studied in various pathological conditions. Its etiology is diverse.

This review focuses on the oxidative processes that occur mainly in the vasculature and lead to vascular dysfunction.

### Vascular Oxidative Stress

Regulation of vascular tone is critical for the homeostatic function of blood vessels and blood supply to peripheral organs. In physiological conditions, maintenance of appropriate endothelial function provides vasorelaxant properties through release of vasoactive substances (Sena et al., 2013). An imbalance between production of vasoprotective and vasorelaxant factors and vasoconstrictor substances by the endothelium is a hallmark of endothelial dysfunction, which precedes numerous vascular pathologies (Sena et al., 2013). In this context, oxidative stress has a crucial role in the initiation and progression of endothelial dysfunction and vascular disease affecting several cells in the vascular wall (Giacco and Brownlee, 2010; Sena et al., 2013; Rodriguez-Porcel et al., 2017).

In addition, vascular oxidative stress promotes systemic inflammation via immune activation. Activated immune cells migrate into the vasculature, and release several factors including ROS, metalloproteinases, cytokines, and chemokines that promote dysfunction and cause vascular damage promoting vasoconstriction and remodeling of blood vessels (Zhou et al., 2017; Norlander et al., 2018). Vascular remodeling, stiffness, structural elastin abnormalities, and increased oxidative stress are hallmarks of vascular damage in hypertension (Martínez-Revelles et al., 2017).

### Endothelium

Endothelial cells regulate vascular tone having a crucial role in the control of organ vascular resistance. These cells, through their secretome, influence vascular smooth muscle cells (VSMCs) and circulating cells such as platelets and monocytes (Sena et al., 2013; Rodriguez-Porcel et al., 2017).

The arterial endothelium is subjected to various injurious stimuli such as oscillatory shear stress, disturbed turbulent flow and oxidative stress among others. The major ROS produced in response to several stimuli (such as hyperglycemia, hyperlipidemia, and hypertension) is superoxide anion (${\text{O}}_{2}^{•-}$) that quickly combines with NO to produce peroxynitrite decreasing NO bioavailability and leading to endothelial dysfunction (Sena et al., 2008, 2011). In response, endothelial cells became activated, produce vasoconstrictor agents (thromboxane A2, endothelin-1, or prostaglandin H2) and an inflammatory response is initiated. The endothelium starts expressing adhesion molecules and secretes chemokines such as chemokine (C-C motif) ligand 2 (CCL2) to attract immune cells (Sena et al., 2013). The increased expression of chemokines and proteases in endothelial cells creates a vicious circle perpetuating the inflammatory response (Campbell et al., 2015). During prolonged vascular inflammation, several changes such as an increment in apoptotic cells, remodeling of the extracellular matrix, breakdown of elastic lamella, and endothelial dysfunction, emerge in atheroprone vessels and accelerate atherosclerosis-related complications (Weber and Noels, 2011). Dysfunctional endothelium is crucial to initiate vascular dysfunction leading to several pathologic conditions including macro (atherosclerosis) and microvascular diseases.

### Vascular Smooth Muscle Cells

Vasoactive substances exert their vasorelaxant or vasoconstrictive properties through effects on receptors of VSMCs, which are central for the regulation of vascular tone. These substances derive from endothelial cells, vasoactive nerves, and perivascular tissue (Giacco and Brownlee, 2010; Majesky, 2015).

Vascular smooth muscle cells can also be sources of ROS promoting oxidative stress (Lim and Park, 2014). Various stimuli including increased cyclic stretch can promote oxidative stress in VSMCs. It was recently described that lysyl oxidase (LOX), an elastin crosslinking enzyme, is as a novel source of vascular ROS. LOX-derived ROS activate p38 mitogen-activated protein kinase critically influencing elastin structure and vessel stiffness in hypertension (Martínez-Revelles et al., 2017).

Activation of oxidative stress and inflammation occurs via receptors leading to changes in the balance between vasodilators and vasoconstrictors affecting vascular tone and ultimately lead to vascular dysfunction (Bhatt et al., 2014; Lim and Park, 2014). Vascular ROS effects are mediated through redox-sensitive signaling pathways. ROS regulate protein kinases, phosphatases, mitogen-activated protein kinases, and transcription factors; playing an important role as modulators of [Ca^2+^]_i_, rho-associated coiled-coil protein kinase (ROCK), and the contractile machinery. Oxidative stress will activate protein kinase C (PKC) leading to the activation of nicotinamide adenine dinucleotide phosphate (NADPH) oxidases and in turn increment oxidative stress. PKC activation will phosphorylate and activate multiple mechanisms that ultimately promote VSMCs contraction. Increased Nox-derived ROS generation enhances calcium signaling, up-regulates ROCK and modulates the actin cytoskeleton, thereby promoting vascular contraction and increasing vascular tone. PKC and ROCK activity play a role in exacerbated vasoconstriction associated with vascular dysfunction (Liu and Khalil, 2018).

### Adventitia and Perivascular Adipose Tissue

The adventitia surrounds the tunica media containing many different cell types in close continuity with perivascular tissue (Majesky, 2015), particularly in large arteries, influencing both endothelial (through vasa vasorum) and VSMCs. In this context, perivascular adipose tissue (PVAT) is an important regulator of vasculature, with much more than a supportive and mechanical role it has also endocrine and paracrine functions. Visceral adipose tissue is a known source of adipokines but PVAT is also an active producer of both adipokines and inflammatory cytokines (Omar et al., 2014).

Under physiological conditions, PVAT has anti-contractile and anti-inflammatory properties that protect blood vessels. Recent studies have suggested that aortic PVAT is protective for endothelial dysfunction in hypercholesterolemic LDL receptor knockout mice. Elevated eNOS-derived NO production in aortic PVAT of this animal model is an adaptive mechanism that may protect endothelial function and maintain normal endothelium-dependent relaxation in the early stages of atherosclerotic disease (Baltieri et al., 2018).

In pathologic conditions such as obesity and atherosclerosis, PVAT changes its phenotype and can contribute to vascular oxidative stress because they have an increment in NADPH oxidases in adipocytes with increase activity in obesity-related conditions (Gao et al., 2006; Gil-Ortega et al., 2014; Padilla et al., 2015). In fact, an increment in ROS has been previously described (DeMarco et al., 2010; Salgado-Somoza et al., 2010; Meijer et al., 2011; Sacks and Fain, 2011). In addition, oxidative stress in adipocytes subsequently stimulates recruitment of immune cells (Chatterjee et al., 2009). Perivascular adipocytes also express angiotensinogen (Serazin et al., 2004) and PVAT produces and releases angiotensin II and related peptides (Bujak-Gizycka et al., 2007).

In obesity, PVAT displays hypoxia, inflammation, and oxidative stress culminating in a dysregulated production/secretion of adipokines and cytokines and leading to PVAT changes toward a proinflammatory phenotype. In animal models and in patients with obesity, PVAT phenotype has been described by up-regulation of pro-inflammatory adipokines/cytokines (e.g., leptin, interleukin-6, tumor necrosis factor-α) and down-regulation of anti-inflammatory adipokines/cytokines (e.g., adiponectin and Interleukin-10) (Henrichot et al., 2005; Chatterjee et al., 2009; Marchesi et al., 2009; Ketonen et al., 2010; Meijer et al., 2011; Aghamohammadzadeh et al., 2016; Xia et al., 2016; Sena et al., 2017). In addition, an increment in esterified fatty acids has also been observed (Ghorbani et al., 1997).

The oxidative stress and inflammation in PVAT have a major impact on endothelial function (Wimalasundera et al., 2003; Payne et al., 2010; Sena et al., 2017), vascular stiffness (Tsioufis et al., 2007; Wu et al., 2016), smooth muscle migration (Barandier et al., 2005) and ultimately lead to vascular disease (Marchesi et al., 2009; Ketonen et al., 2010; Ma et al., 2010; Almabrouk et al., 2014; Gil-Ortega et al., 2014; Omar et al., 2014; Molica et al., 2015) reinforcing the “vasocrine” effects of PVAT in obesity-related diseases.

### Sources of Reactive Oxygen Species

**FIGURE 1:**
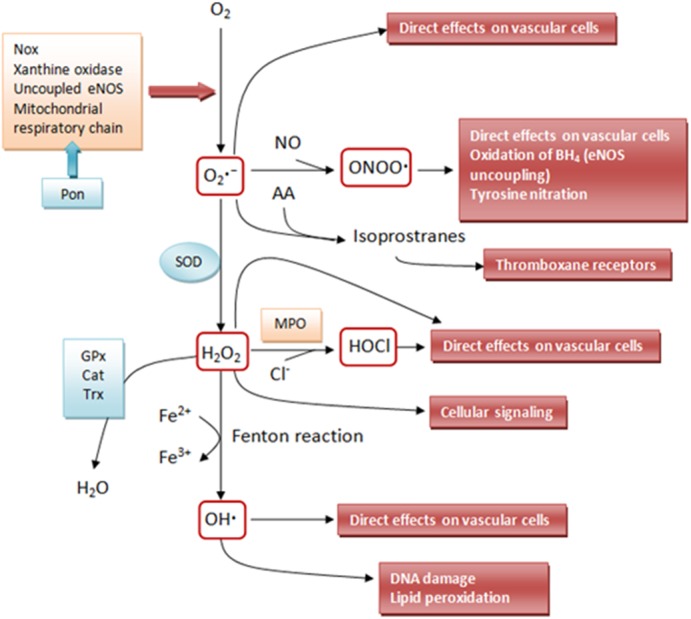
Schematic outline of the interrelationships between some of the more relevant reactive oxygen species (ROS) that affect the vascular wall. Superoxide (${\text{O}}_{2}^{•-}$) is produced from molecular oxygen (O_2_) by different sources such as nicotinamide adenine dinucleotide phosphate – NADPH oxidase (Nox), mitochondrial respiratory chain, xanthine oxidase, uncoupled endothelial nitric oxide synthase (eNOS) and lipoxygenases. Superoxide can directly affect vascular cells but can also be converted by superoxide dismutases (SOD) to hydrogen peroxide (H_2_O_2_). H_2_O_2_ can undergo spontaneous conversion to hydroxyl radical (OH∙, extremely reactive – attacks most cellular components) in the presence of iron (Fe^2+^) via the Fenton reaction. H_2_O_2_ produces direct effects on the vascular wall or can be detoxified via glutathione peroxidase (GPx), catalase (Cat), or thioredoxin (Trx) peroxidase to H_2_O and O_2_. Superoxide can also react with nitric oxide (NO) or arachidonic acid to form peroxynitrite (ONOO^⋅-^) or isoprostanes, respectively. In addition to other signaling effects, H_2_O_2_ can activate Nox, resulting in further production of superoxide. The enzyme myeloperoxidase (MPO) can use H_2_O_2_ to oxidize chloride to the strong-oxidizing agent hypochlorous acid (HOCl). HOCl can chlorinate and thereby inactivate various biomolecules including lipoproteins and the eNOS substrate L-arginine. Besides HOCl generation, myeloperoxidase can oxidize (and thus inactivate) NO to nitrite (${\text{NO}}_{2}^{-}$) in the vasculature. Paraoxonase (PON) isoforms 2 and 3 can prevent mitochondrial O_2_∙– generation (Adapted with modifications from De Silva and Faraci, 2017).

In biological systems most ROS are generated from mitochondria (Cadenas and Davies, 2000). Mitochondrial dysfunction is an important cause in the development and progression of several diseases: energy surplus and oxidative stress causes the mitochondrial dysfunction fostering ROS production and oxidative stress. Other sources include NADPH oxidase, xanthine oxidase, cytochrome P450, uncoupled endothelial nitric oxide synthase (eNOS enzyme produces ${\text{O}}_{2}^{•-}$ instead of NO), myeloperoxidases and lipoxygenases (Madamanchi et al., 2005; Santilli et al., 2015) (Figure 1).

## Pro-Oxidant Pathways

### NADPH Oxidase

NADPH oxidases (Nox) are a crucial contributor to oxidative stress in vascular cells, including endothelial cells, VSMCs, fibroblasts, and perivascular adipocytes (Griendling et al., 2000; Cai et al., 2003). Nox expression and activity are closely linked with clinical risk factors for atherosclerosis (Guzik et al., 2000). There are at least seven variations of the Nox, characterized by their different catalytic subunits (Guzik and Touyz, 2017).

In blood vessels, Nox1 expression is residual under basal conditions and increases considerably after stimuli (Lassègue and Clempus, 2003). Nox2 generates both ${\text{O}}_{2}^{•-}$ and H_2_O_2_ directly affecting both NO bioavailability and contractile properties of the vasculature (Judkins et al., 2010; Guzik and Touyz, 2017). Nox4 is expressed in all vascular and some perivascular cell types. Nox4 possesses vasorelaxant properties via eNOS activation. It predominantly produces H_2_O_2_ and only small amounts of ${\text{O}}_{2}^{•-}$. In the context of atherosclerosis, Nox4 expression appears to exert vasoprotective effects (Ellmark et al., 2005; Guzik and Touyz, 2017). Nox4 was shown to exert both a beneficial as well as detrimental effect (Lener et al., 2009; Kozieł et al., 2013), depending on the cell context and stimuli that influence its activity. Indeed, Nox4 is a source of ROS, which changes the redox-state of numerous proteins, and further research is needed to clarify its role in the vasculature. Nox 5 is also expressed in blood vessels. It is a Nox sensitive calcium isoform that produces ${\text{O}}_{2}^{•-}$ (Montezano et al., 2018). Nox 5 is a pro-contractile Nox isoform important in redox-sensitive contraction. It was recently described a novel function for vascular Nox5, linking calcium and ROS to the pro-contractile molecular machinery in VSMCs (Montezano et al., 2018). Further studies are necessary to clarify Nox5 functions.

The renin-angiotensin system also stimulates NADPH oxidase activity contributing to oxidative stress, endothelial dysfunction, and structural vascular changes typical of hypertension and atherosclerosis (Dzau, 1987; Heart Outcomes Prevention Evaluation Study Investigators et al., 2000; Guzik and Touyz, 2017).

### The Mitochondrial Respiratory Chain

Mitochondrial oxidative phosphorylation produces ${\text{O}}_{2}^{•-}$, which is transformed to H_2_O_2_ by the manganese-dependent superoxide dismutase and subsequently to water by glutathione peroxidase 1 (Boveris et al., 1976). Under pathological conditions, due to insufficient ROS detoxification or excessive ROS production mitochondrial oxidative stress arises (Madamanchi et al., 2005; Yu et al., 2012; Murphy et al., 2016). Several diseases including atherosclerosis in human have been linked with mitochondrial dysfunction and subsequent oxidative stress (Corral-Debrinski et al., 1992).

### Xanthine Oxidase

Endothelium dysfunction is linked with an increment in endothelial xanthine oxidase expression (Landmesser et al., 2002; Spiekermann et al., 2003) due to the increased production of superoxide and H_2_O_2_. The activity of this enzyme is increased in patients with coronary artery disease (Landmesser et al., 2007) and inhibitors of this enzyme decrement endothelial dysfunction in both humans and animal models (Guthikonda et al., 2003; Schröder et al., 2006; Nomura et al., 2014).

### Uncoupled eNOS

Uncoupled eNOS generates ${\text{O}}_{2}^{•-}$ instead of NO due to low levels of its cofactor tetrahydrobiopterin (BH_4_) or its substrate L-arginine (Alp and Channon, 2004). ROS, in particular peroxynitrite, promote eNOS “uncoupling” (Sena et al., 2013; Santilli et al., 2015). Superoxide reacts with NO forming peroxynitrite that further oxidizes BH_4_ to dihydrobiopterin (BH_2_), creating a vicious circle and more eNOS uncoupling (Li and Forstermann, 2014). Under physiological conditions, PVAT prevents eNOS uncoupling (Ebrahimian et al., 2009).

### Myeloperoxidase

Myeloperoxidase (MPO) is an enzyme that belongs to the mammalian heme peroxidase superfamily, present in polymorphonuclear neutrophils and in monocytes/macrophages (Lefkowitz et al., 2010). MPO produces various compounds with pro-oxidant properties contributing to oxidative stress by oxidizing LDL and lowering NO bioavailability (Pitanga et al., 2014). This enzyme is involved in the formation of products derived from the oxidation of arachidonic acid that are involved in the inflammatory response and in lipid peroxidation (Zhang et al., 2002; Kubala et al., 2010). In addition, MPO promotes atherogenesis through the production of modified subtypes of LDL and HDL lipoproteins (Daugherty et al., 1994; Nicholls and Hazen, 2009; Kettle et al., 2014).

### Lipoxygenases

Lipoxygenases (LOXs) are intracellular enzymes that peroxidize polyunsaturated fatty acids into bioactive lipids with a potential important role in the pathogenesis of atherosclerosis. LOXs, in particular 5-LOX and 12/15 LOX were found to be overexpressed in advanced atherosclerotic lesions. 5-LOX converts arachidonic acid into leukotriene B4, a potent chemo-attractant and leukocyte activator (Rådmark et al., 2015). However, inconclusive data were obtained with respect to the pathophysiological relevance of this leukotriene signaling in atherosclerosis. Thus, more studies are necessary to clarify this matter (Kuhn et al., 2015).

### Antioxidant Defenses

In the vascular wall, the primary antioxidant defense systems to neutralize ROS production are enzymatic detoxifiers such as superoxide dismutases (MnSOD, CuZnSOD, EcSOD), catalase, glutathione peroxidase, paraoxonase, thioredoxin peroxidase, and heme oxygenases (Santilli et al., 2015). In addition, the transcription factor nuclear factor erythroid-2 related factor 2 (Nrf2) has also been shown to play a key role in establishing a cellular anti-oxidant defense mechanism against oxidative stress (Bryan et al., 2013; Lee, 2017) and is consider an important therapeutic target to manage vascular dysfunction (Förstermann, 2008).

## Therapeutics

Current pharmacological approaches for prevalent diseases, such as obesity, diabetes, and cardiovascular diseases are limited in efficacy. Many studies with antioxidants have proven unsuccessful in clinical trials (Steinhubl, 2008). Hence, the search for new therapies is very important an emergent in order to improve the health status and increase lifespan of the patients.

### Lifestyle Approaches

Lifestyle interventions are capable of reducing body weight through an increment in physical exercise and a reduction in caloric intake. Weight loss by calorie restriction and/or exercise can improve the global health state reducing oxidative stress (Imayama et al., 2012).

### Mitochondrial-Targeted Therapies

An important and potentially useful therapeutic approach for diseases associated with an increment in oxidative stress is to target antioxidants (as ubiquinol or α-tocopherol) to the mitochondria (Milagros Rocha and Victor, 2007; Murphy and Smith, 2007) with lipophilic cations such as mitoquinone (MitoQ) or MitoE2 (Murphy and Smith, 2007; Smith et al., 2008). However, some studies revealed that MitoQ may be prooxidant and proapoptotic because its quinone group can participate in redox cycling and superoxide production. In light of these results, studies using mitoquinone as an antioxidant should be interpreted with caution (Doughan and Dikalov, 2007).

In addition, enzymatic systems or cell-permeable cationic peptides (Szeto-Schiller peptides) directed to the mitochondria can also be an alternative therapeutic approach (Szeto, 2006).

The mitochondrial protein p66^Shc^ is fundamental in the homeostasis of mitochondria. Targeting this adaptor is another form of decreasing mitochondrial oxidative stress. P66^Shc^ leads to ROS generation (Camici et al., 2015) and its inactivation may be essential in oxidative stress related diseases (Francia et al., 2004). Previous studies have demonstrated that the expression of p66^Shc^ is reduced by SIRT1 activation, which in turn decrements oxidative stress and endothelial dysfunction (Chen et al., 2013). SIRT mimetics are therefore promising tools to mitigate vascular disease progression.

### Nrf2 Activators

Nrf2 is a transcription factor ubiquitously expressed in various tissues (including the vasculature) of human and animal models (Pereira et al., 2017; Suzuki and Yamamoto, 2017). It regulates the expression of several antioxidant and detoxification enzymes by binding to upstream antioxidant response elements (Ungvari et al., 2011; Juurlink, 2012).

This transcriptional factor is crucial in the prevention of oxidative stress related-diseases (Kobayashi and Yamamoto, 2006; Lee, 2017). Nrf2 interacts with antioxidant response element sequences of genes coding for antioxidant enzymes include γ-glutamyl cysteine ligase, NAD(P)H quinone oxidoreductase-1, glutathione *S*-transferase, heme oxygenase-1, uridine diphosphate glucuronosyl transferase, superoxide dismutase, catalase, and glutathione peroxidase-1 having a major role in cellular responses to oxidative stress (Alam et al., 1999).

Nrf2 activators can act through different mechanisms (Niture et al., 2014): they can directly prevent the interacting of Nrf2 with the Nrf2-binding site of Kelch-ECH-associated protein-1 (KEAP1) (as, for instance, ML334) (Jiang et al., 2016), they enhance transcription of Nrf2-targeted antioxidant genes (as berberine) (Imenshahidi and Hosseinzadeh, 2016) or they specifically and reversibly increment Nrf2 half-life (as MG-132) (Dreger et al., 2009). Other compounds promote the release of Nrf2 from KEAP1 through interaction with its cysteine thiol residues (as sulforaphane) (Hong et al., 2005).

### Nox Inhibitors and Recouplers of eNOS

Triazolopyrimidines are efficient and selective inhibitors of NADPH oxidase activity. They specifically inhibit NADPH oxidase-derived ROS *in vitro* (ten Freyhaus et al., 2006; Drummond et al., 2011; Santilli et al., 2015) and have great potential in the field of atherosclerosis.

GKT137831, an inhibitor of NOx 1 and Nox4, reduces oxidative stress and diabetic vasculopathy (Gray et al., 2017) and is currently under clinical trial. GLX351322 has been suggested as a therapeutic approach in type 2 diabetes through its selective inhibition of NOX4 (Anvari et al., 2015). In addition, rutin, a glycoside of quercetin, exhibits anti-oxidant and anti-inflammatory properties protecting endothelial dysfunction through inhibition of NOx4 and the ROS-sensitive NLRP3 inflammasome (Wang et al., 2017).

The aldehyde dehydrogenase 2 and its activator Alda-1 are novel molecules capable of inhibiting toxic aldehydes, decreasing oxidative stress and NADPH oxidase activity. These molecules are able to reverse mitochondria dysfunction having a potential role in atherogenesis (Stachowicz et al., 2014; Yang et al., 2018). Nebivolol inhibits the activity of NADPH oxidase and, in addition, reverses eNOS uncoupling (Munzel and Gori, 2009).

Inhibiting eNOS uncoupling is another strategy capable of reducing ROS. Among the drugs used in the clinical practice, inhibitors of the renin–angiotensin–aldosterone system, statins, metformin and pentaerythritol tetranitrate have been described to prevent or reverse eNOS uncoupling. Other compounds, such as resveratrol, sepiapterin, BH_4_, folic acid, α-lipoic acid and AVE3085, improve endothelial function through eNOS recoupling although further studies are needed to validate these compounds (Li and Forstermann, 2014; Xia et al., 2017). Recently, thioredoxin was shown to reverse age-related hypertension and arterial stiffness by improving vascular redox and restoring eNOS function (Hilgers et al., 2017).

### Targeting Hyperglycemic Memory

Inhibiting hyperglycemic memory concomitantly with a reduction in oxidative stress is crucial under diabetic conditions. The hyperglycemic memory needs to be reduced in diabetes in order to prevent the progression of diabetic complications (Bianchi et al., 2013). Otherwise, this occurrence will result in a vicious cycle continuously producing ROS and leading to vascular dysfunction. Targeting the incretin pathway with dipeptidyl peptidase inhibitors or glucagon-like peptide receptor-1 agonists was able to reduce advanced glycation end products (AGEs) and ROS downstream events that promote cellular damage (Berezin, 2016). Therapies associated with reversion of hyperglycemic memory deserve further investigation in this field and include soluble RAGE/RAGE antagonists, AGE/methylglyoxal inhibitors, S100 inhibitors, targeting vascular protein lysine acetylation (Carrillo-Sepulveda, 2017; Kraakman et al., 2017), among others.

### Anti-diabetic Drugs

Studies both in humans and in animal models revealed that anti-diabetic drugs (such as metformin) are able to decrement oxidative stress and inflammation and ameliorate endothelial function reducing the progression toward atherosclerosis (Sena et al., 2009, 2011; Jenkins et al., 2018). Incretin mimetics, in particular GLP1 agonists and dipeptidyl peptidase-4 (DPP4) inhibitors, are currently anti-diabetic agents with a wide range of beneficial effects that include antioxidant effects. An example is linagliptin, an inhibitor of DPP4 that reduces obesity-related insulin resistance and inflammation by regulating M1/M2 macrophage status (Zhuge et al., 2016). It was recently shown that saxagliptin, an inhibitor of DPP4, prevented coronary vascular stiffness through a mechanism that involved a decrement in AGEs, NF-κB, and nitrotyrosine levels in aortic-banded mini swine (Fleenor et al., 2018).

In obesity, diabetes and hypertension mineralocorticoid receptor antagonism improves endothelial function and seems to be a mediator of the switch from vascular health to disease. In endothelial cells, the mineralocorticoid receptor exerts a protective role on endothelial function. In the presence of cardiovascular risk factors, endothelial mineralocorticoid receptor contributes to endothelial dysfunction through NOX activation, eNOS uncoupling, increased epithelial sodium channel expression, and ICAM1/VCAM1-mediated inflammation. Further studies are necessary to clarify these mechanisms (Bakris et al., 2015; Davel et al., 2017).

### PCSK-9 Inhibitors

PCSK9 inhibitors are monoclonal antibodies that bind to and inactivate proprotein convertase subtilisinkexin 9 (PCSK9), a liver enzyme that promotes the lysosomal degradation of LDL receptors in hepatocytes thus increasing the number of LDL receptors in the membrane and LDL-cholesterol uptake. In recent studies, evolocumab for 52 weeks significantly decreased the level of vitamin E in LDL-C and increased vitamin E level in HDL (Blom et al., 2015) revealing antioxidant properties (Sabatine et al., 2017).

### Other Pharmacological Approaches

Serotonin in peripheral blood reflects oxidative stress and plays an important role in atherosclerosis highlighting the novel anti-atherothrombotic strategy to mitigate vascular disease (Sugiura et al., 2016). Tropisetron, a 5-HT3 receptor antagonist, can attenuate early diabetes through calcineurin inhibition and by suppressing oxidative stress and some inflammatory cytokines in streptozotocin-induced diabetic rats (Barzegar-Fallah et al., 2015).

More recently, cell-permeable peptides mimicking the kinase inhibitory region of suppressor of cytokine signaling- 1 (SOCS1) regulatory protein emerged as an important antioxidant and anti-inflammatory strategy to limit the progression of diabetic complications (Lopez-Sanz et al., 2018). SOCS-targeted therapies have recently been suggested as potential therapeutic approaches in atherosclerosis and deserve further investigation.

Other approach includes the inhibition of selenoprotein P (SeP). SeP is a liver derived secretory protein that promotes insulin resistance (Misu et al., 2010) and is upregulated in the liver of type 2 diabetic patients. This hepatokine downregulates the metabolic switch, AMP-activated protein kinase (AMPK) (Misu et al., 2010). Recent studies have suggested that SeP regulates cellular metabolism and the development of vascular diseases (Ishikura et al., 2014; Cetindaǧlı et al., 2017; Mita et al., 2017; Kikuchi et al., 2018). SeP promotes vascular smooth cell proliferation through increased oxidative stress and mitochondrial dysfunction in an autocrine/paracrine manner. Sanguinarine, an orally active small molecule, reduces SeP expression and smooth muscle proliferation, and ameliorates pulmonary arterial hypertension in mice and rats. Thus, it seems that SeP could be a novel and realistic therapeutic target (Kikuchi et al., 2018).

The clinical benefit of these compounds awaits further confirmation. Moreover, novel nanotherapeutic approaches are being developed to target oxidative stress and inflammation (Wang et al., 2018).

Traditional medicinal plants have been used by several civilizations through the years contributing to the notion that natural products are relevant sources of new pharmaceutical compounds. Technological advances are now capable of unravel the value of natural products as novel sources for new drug discovery including their role as antioxidants.

## Conclusion

Vascular oxidative stress promotes endothelial dysfunction and atherosclerosis progression. In the vasculature, several sources promote an increment in oxidative stress.

Preventing vascular oxidative stress and incrementing NO bioavailability may represent the future therapeutic strategy to mitigate the cardiovascular burden and reduce associated risk factors.

## Author Contributions

CS wrote the article. AL did bibliographic research. LA did bibliographic research. RS revised the manuscript. GP revised the manuscript.

## Conflict of Interest Statement

The authors declare that the research was conducted in the absence of any commercial or financial relationships that could be construed as a potential conflict of interest.
